# Supplementary material for: Choice-relevant information transformation along a ventrodorsal axis in the medial prefrontal cortex
Source: Nat Commun. 2021 Aug 10;12:4830. doi: 10.1038/s41467-021-25219-w (PMC8355277; doi:10.1038/s41467-021-25219-w)
Supplement: Supplementary file 2 — Reporting Summary [file 41467_2021_25219_MOESM2_ESM.pdf]

nature portfolio

Corresponding author(s): Maisson, David

Last updated by author(s): Jul 22, 2021

Reporting Summary

Nature Portfolio wishes to improve the reproducibility of the work that we publish. This form provides structure for consistency and transparency in reporting. For further information on Nature Portfolio policies, see our Editorial Policies and the Editorial Policy Checklist.

Statistics

For all statistical analyses, confirm that the following items are present in the figure legend, table legend, main text, or Methods section.

n/a	Confirmed	
☐	☒	The exact sample size (n) for each experimental group/condition, given as a discrete number and unit of measurement
☐	☒	A statement on whether measurements were taken from distinct samples or whether the same sample was measured repeatedly
☐	☒	The statistical test(s) used AND whether they are one- or two-sided<br>*Only common tests should be described solely by name; describe more complex techniques in the Methods section.*
☐	☒	A description of all covariates tested
☐	☒	A description of any assumptions or corrections, such as tests of normality and adjustment for multiple comparisons
☐	☒	A full description of the statistical parameters including central tendency (e.g. means) or other basic estimates (e.g. regression coefficient) AND variation (e.g. standard deviation) or associated estimates of uncertainty (e.g. confidence intervals)
☐	☒	For null hypothesis testing, the test statistic (e.g. F, t, r) with confidence intervals, effect sizes, degrees of freedom and P value noted<br>*Give P values as exact values whenever suitable.*
☒	☐	For Bayesian analysis, information on the choice of priors and Markov chain Monte Carlo settings
☒	☐	For hierarchical and complex designs, identification of the appropriate level for tests and full reporting of outcomes
☐	☒	Estimates of effect sizes (e.g. Cohen's d, Pearson's r), indicating how they were calculated

Our web collection on statistics for biologists contains articles on many of the points above.

Software and code

Policy information about availability of computer code

Data collection	The custom Matlab code used to generate the Risky Choice Task in this study will be made available upon reasonable request of the corresponding author.
Data analysis	No new methological approaches were developed for this study. All functions used for analyzing the data as described in the methods section of the manuscript are standard implementations in Matlab 2017a. Code-specific to this dataset has been archived and can be requested from the authors.

For manuscripts utilizing custom algorithms or software that are central to the research but not yet described in published literature, software must be made available to editors and reviewers. We strongly encourage code deposition in a community repository (e.g. GitHub). See the Nature Portfolio guidelines for submitting code & software for further information.

Data

Policy information about availability of data

All manuscripts must include a data availability statement. This statement should provide the following information, where applicable:

- Accession codes, unique identifiers, or web links for publicly available datasets
- A description of any restrictions on data availability
- For clinical datasets or third party data, please ensure that the statement adheres to our policy

These data continue to be in use within the lab and among collaborators for subsequent follow-up analyses. In order to protect our agreements with collaborators, and the projects and manuscripts currently on-going, we are not yet ready to make the entire dataset open-access at this time. This is why we are fully prepared to provide appropriate segments of the data upon reasonable request. The specific aggregate of the data used in this manuscript can be requested as-is. Source Data, the relevant raw data used to generate each figure, are available as a Source Data file on Dryad (https://doi.org/10.5061/dryad.18931zcxv).

Field-specific reporting

Please select the one below that is the best fit for your research. If you are not sure, read the appropriate sections before making your selection.

- [x] Life sciences
- [ ] Behavioural & social sciences
- [ ] Ecological, evolutionary & environmental sciences

For a reference copy of the document with all sections, see nature.com/documents/nr-reporting-summary-flat.pdf

Life sciences study design

All studies must disclose on these points even when the disclosure is negative.

Sample size	A sample size of 2 animals per recorded area is the standard in neuroeconomic primate research (Padoa-Schioppa and Assad, 2006; Strait et al., 2014; Grattan and Glimcher, 2014; Yoo et al. 2020).
Data exclusions	No data were excluded from our analyses.
Replication	Replication, as a methodology, is not applicable to this type of research, as there are no exogenous experimental manipulations beyond that of the behavior. The extent to which there is any possible replication is that the task itself has been used repeatedly in the lab and published on numerous times such that the same behavioral patterns are continually recapitulated. We have indicated as much both within the Results section and the methods section, including citations that have used the same task and behavior.
Randomization	There are no control or experimental groups built into the design of this study, as no experimental manipulations were introduced beyond the structure of the Risky Choice Task; which was performed the same way across subjects as stipulated in the Methods section.
Blinding	No blinding was necessary, as there was no group allocation in the experimental design.

Reporting for specific materials, systems and methods

We require information from authors about some types of materials, experimental systems and methods used in many studies. Here, indicate whether each material, system or method listed is relevant to your study. If you are not sure if a list item applies to your research, read the appropriate section before selecting a response.

Materials & experimental systems

n/a	Involved in the study	
x		Antibodies
x		Eukaryotic cell lines
x		Palaeontology and archaeology
	x	Animals and other organisms
x		Human research participants
x		Clinical data
x		Dual use research of concern

Methods

n/a	Involved in the study	
x		ChIP-seq
x		Flow cytometry
x		MRI-based neuroimaging

Animals and other organisms

Policy information about studies involving animals; ARRIVE guidelines recommended for reporting animal research

Laboratory animals	Adult (age 4-8) male macaca mulatta (rhesus macaques) were used in this study.
Wild animals	No wild animals were used in this study.
Field-collected samples	No field-collected sample were used in this study.
Ethics oversight	The University Committee on Animal Resources at the University of Rochester and University of Minnesota approved all animal procedures. Animal procedures were designed and conducted in compliance with the Public Health Services Guide for the Care and Use of Animals.

Note that full information on the approval of the study protocol must also be provided in the manuscript.
